# Time course from cochlear implant surgery to non-use for congenitally deaf recipients implanted as children over ten years ago

**DOI:** 10.3389/fresc.2023.1283109

**Published:** 2023-12-01

**Authors:** Catherine Killan, Han Cao, Angela Cordingley, David Strachan

**Affiliations:** ^1^ NIHR Nottingham Biomedical Research Centre, Hearing Sciences, Mental Health and Clinical Neurosciences, School of Medicine, University of Nottingham, Nottingham, United Kingdom; ^2^ Yorkshire Auditory Implant Service, Bradford Teaching Hospitals Foundation NHS Trust, Bradford, United Kingdom

**Keywords:** cochlear implant, non-use, congenital deafness, time, duration, risk factors, sign language

## Abstract

**Objective:**

To determine the time-course from first cochlear implantation to non-use, to characterise non-users' receptive and expressive communication, and document known risk factors for inconsistent use, for congenitally deaf non-users of cochlear implants implanted as children at least ten years ago.

**Methods:**

Retrospective service evaluation. All congenitally deaf patients who received a first cochlear implant as children at least ten years ago at a regional service, and were currently non-users, were identified. They were characterised in terms of ages at implantation and non-use, known risk factors for inconsistent CI use or CI non-use, and outcome measures were the Meaningful Auditory Integration Scale (MAIS) and Meaningful Use of Speech Scale (MUSS) scores.

**Results:**

Seventeen patients met the inclusion criteria. They were implanted from 1990 to 2006. Median age at implantation was 4 years (range: 2–11), median age at non-use was 17 years (range: 9–31), and median duration of use was 8.5 years (range: 4–25). All used sign or gesture as their primary expressive and receptive communication modes. In addition, each child had at least one other known risk factor for inconsistent CI use. At 3 years post-implantation, mean Parent-rated MAIS scores were 76.5% (*N* = 14), and mean MUSS scores were 43.1% (*N* = 9).

**Discussion:**

This cohort included cases where CI use was rejected following longer periods of time than previously reported, highlighting a need for long-term support, particularly around the ages of life transitions. Studies conducted when the earliest cohort of paediatric CI users were younger, and studies reliant on parent or patient reports, may under-estimate long-term non-use rates. No non-users were identified among congenitally-deaf children implanted 10–15 years ago. Further research is warranted to explore relationships between risk factors, including communication mode, and non-use to inform expectation setting and candidacy selection.

## 1. Introduction

Cochlear implants (CIs) are a safe, cost-effective intervention for children born with severe-to-profound hearing loss (1). However, a minority of children who receive CIs later choose to become device non-users (2–5). It is important to minimize the occurrence of CI non-use. Limited use is associated with stress for both users and their parents (5, 6). Non-use reduces the cost-effectiveness of the intervention and therefore could negatively influence the commissioning of CIs by healthcare systems (4, 7). Understanding children who became non-users could inform candidacy selection and service provision to minimize the chance that children in the future will reject their CIs. Optimizing the chances of successful CI use requires both well-informed selection of appropriate candidates and providing enough support to users. An understanding of the time-course from surgery to non-use can equip CI services to make informed decisions on resource allocation to provide adequate long-term support.

Various factors have been identified as being associated with a higher risk of either inconsistent, less than full-time, or complete non-use of CIs by children. Several of the risk factors listed below were identified by multiple studies and examples include: older age at implantation (4); the presence of additional needs (2, 8); technical, medical and surgical complications (9); psycho-social factors including peer pressure (10); lower maternal education (8); financial barriers to accessing care (9); complex family issues (3); poor attendance at appointments (5); inconsistent device use over time (3), reliance on signed communication at home and or in education (4), poor expressive spoken language outcomes (3), and there being no perceived benefit from implantation (11). The varied nature of these risk factors highlights the need for multidisciplinary care of CI users. The presence of risk factors such as age at implantation and communication mode suggests that the population this special edition is focussed on, congenitally deaf children who received their CIs in the earlier years of their availability, might be the most vulnerable to CI non-use in the UK. This population did not all have access to early diagnosis or intervention. In some cases they were implanted prior to understanding or acceptance of the technology among non-specialists who were supporting them in their educational settings. Even among CI specialists, the importance of early intervention was not yet fully understood. At the time of writing, many children globally do not have access to neonatal hearing screening or specialist rehabilitation services (12), so an understanding of people who received their CIs as congenitally deaf children over a decade ago remains relevant, both for their own care and the future management of other deaf children.

For service providers to plan the rehabilitation support needed for those at risk of non-use, it is necessary to understand the time-course over which non-use can happen. Several studies have noted that the proportion of CI users within a service reporting full-time use declines over time (4, 9) and, because of the recent introduction of CIs in comparison to the life span of many recipients, it is not yet known for how long the risk of non-use remains. In studies of non-users the follow-up time increases with later publication date, for example from up to 3 years (13), 7 years (2, 3), 14 years (8) to 30 years (11). Several studies have provided rich information, using either datalogging technology which needs to be actively downloaded from speech processors (8), or from patient or parent reports (3, 9, 11) meaning it was not possible to include implanted patients who had lost contact with the CI service. Such study designs risk not including patients most likely to be device non-users or, if reporting outcomes only for child CI recipients who are still children at the time of analysis, missing the later onset of CI non-use during adult life.

There are benefits in services sharing long-term data on all non-users. The information can help other clinics plan long-term care strategies, inform future candidate selection, optimize the cost-effectiveness of cochlear implantation, and minimize raising unrealistically high expectations in families whose children might not benefit. Knowledge around the time-course from implantation to non-use could reveal stages in children's development when they might be most vulnerable to becoming non-users. Documenting the characteristics, including the receptive and expressive communication, of congenitally deaf child CI recipients who became non-users can inform future CI candidacy decisions. Awareness of vulnerable periods during childhood and adolescence, and of the characteristics of this population, could be especially useful for services providing CIs in areas where universal hearing screening is not well established, and centers have fewer years of experience in supporting congenitally deaf children. This study therefore aimed to determine the time-course from first cochlear implantation to non-use, to characterise non-users' receptive and expressive communication, and document known risk factors for inconsistent use, for congenitally deaf non-users of cochlear implants implanted as children at least ten years ago.

## 2. Materials and methods

### 2.1. Study design

This was a retrospective, observational, service evaluation.

### 2.2. Data extraction

The study was conducted at a regional auditory implant service within a National Health Service tertiary care hospital in England. All congenitally deaf patients who were first implanted under 18 years of age, at least ten years ago (from the start of the service in 1989 to June 2013), and who had become device non-users, were identified from a locally maintained database. This included those who had been explanted and those who had a CI *in situ* but had ceased to use their speech processor. Data were extracted by hand from all available records, both paper and electronic, including via retrieval of paper records from off-site archive facilities, by members of the local care team. Congenital deafness was defined as having been born with sensorineural hearing loss meeting the audiometric criteria for cochlear implantation defined in National Institute for Health and Care Excellence technology appraisal guidance 166 (14), i.e., unaided pure tone thresholds of ≥90 dB HL at both 2 and 4 kHz bilaterally. Patients born with better hearing, even if they lost their hearing during the first year of life, were excluded to keep the cohort aligned with the focus of this special edition. Patients meeting the eligibility criteria were included regardless of if they had been implanted at this site or implanted elsewhere and later transferred into the service.

Participant characteristics extracted included calendar year of first implantation, age at first implantation, duration of CI use, age at confirmation of CI non-use, aetiology, implant model, unilateral or bilateral implantation, educational setting, children's primary receptive and expressive communication modes, sex assigned at birth, and ethnicity. Only group summary characteristic data were presented, and details of additional needs or family issues with-held, to prevent any individuals from being identifiable. Date of non-use was defined as the earliest date in the records when it was noted that they no longer used their implant(s) at all, following which there was no record of them resuming CI use. If narrative descriptions of any potential factors contributing to non-use were available these were transcribed, with any potentially identifiable information removed. Risk factors were categorised as: Chronological age ≥3 years at first implantation; Primary communication sign language or gesture; Inconsistent CI use; Technical or medical device issues; Additional needs; Complex family issues; Poor attendance at CI appointments; Child under peer pressure not to use CI; Family perceived no benefit from CI; Financial barriers to accessing CI care. Speech processor datalogging was not available for this cohort during the time period studied and so judgments around consistent CI use had to be made based on subjective reports from children and their parents or teachers. Inconsistent use was defined as there being reports in the medical notes of a child routinely using their speech processor in only certain settings, for example at school but not at home; parent reports of a child e.g., “rarely using” their speech processor; and descriptions of at least one period of weeks or months during which the CI was not used but following which use was resumed. Maternal education was not available within the notes and therefore was not included as a risk factor category. Expressive and receptive communication was not included as a risk factor category for descriptive data, instead scores from the Meaningful Auditory Integration Scale (MAIS) and Meaningful Use of Speech Sounds Scale (MUSS) (15) were extracted where available. These data are classed as parent report measures, but in line with the recommended administration had been collected by specialist Teachers of the Deaf using unstructured probes in conversation with children's parents. Raw scores had been converted to percent correct. A pseudonomised dataset was shared with a researcher for analysis. The study was conducted with the approval of the hospital's research and innovation department.

### 2.3. Analysis

Quantifiable characteristics were analyzed via descriptive statistics. Potential factors influencing non-use were categorised as: Complex family issues; inconsistent appointment attendance; perceived absence of any benefit; peer pressure; additional needs; device issues including partial insertion, partial electrode array function, medical or surgical complications; inconsistent CI use; being aged 3 years or older at first implantation; and financial barriers. Maternal education was not included due to the unavailability of data. The incidence of these risk factors across the whole group, and cumulative incidence of risk factors per participant, were calculated. Children were classed into sub-groups as being younger at non-use (aged less than 12 years) or older at non-use (aged 12 years or older). Percentage parent-reported MAIS and MUSS scores pre-CI, and at 1 and 3 years post-CI activation, were documented for each individual, and changes from pre-CI to 3 years post-CI were compared for the sub-set of children with both sets of data via dependent *t*-tests. To prevent the identification of any individuals, their ages at first CI and non-use were presented in categories not absolute values when linked with cumulative number of risk factors and MAIS and MUSS scores.

## 3. Results

### 3.1. Participant characteristics

Twenty-four patients were identified who had received a first CI under the age of 18 years prior to June 2013 and were non-users at the point of data extraction. In keeping with the focus of this special edition, children with acquired or progressive hearing loss were excluded from full data extraction and analyses, three of whom had onset of hearing loss during the first year of life. Seventeen patients were identified who met the inclusion criteria. Their medical and audiological characteristics are presented in Table 1. Additional needs included syndromes, behavioural and learning difficulties, and physical disabilities. Details and aetiologies are not presented, to ensure confidentiality.

**Table 1:** Medical and audiological characteristics.

	Whole group *N* = 17
Sex assigned at birth	
Male	6 (35%)
Female	10 (59%)
Missing	1 (6%)
Device configuration	
Unilateral CI	16 (94%)
Bilateral CI	1 (6%)
Cochlear implant manufacturer	
Cochlear Corp	
CI22M	4 (24%)
CI24M	8 (47%)
CI24R	1 (6%)
Med-El	
PulsarCi100	1 (6%)
COMBI C40+	2 (12%)
Missing	
Manufacturer missing	1 (6%)
Implantation service	
Implanted within the service	15 (88%)
Implanted outside the service	2 (12%)
Ethnicity	
White British	10 (59%)
Pakistani	7 (41%)
Aetiology	
Maternal Rubella	2 (12%)
Congenital CMV	3 (18%)
Syndromic	1 (6%)
Aetiology unknown	11 (65%)
Additional needs	
Additional needs documented in CI records	5 (29%)
CI status at time of data extraction	
Explanted	2 (18%)
CI *in situ*	15 (88%)

### 3.2. Time-course from surgery to CI non-use

**Figure 1:**
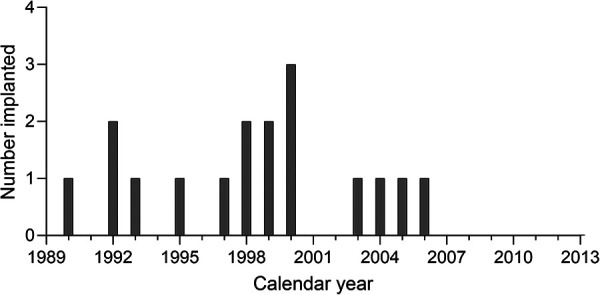
Distribution of calendar year of first implantation.

Any congenitally deaf patients implanted as children prior to June 2013 were potentially eligible for inclusion. However, all the non-users identified were implanted during 1990–2006 inclusive. Figure 1 shows the distribution of year of first cochlear implantation for the whole group.

**Figure 2:**
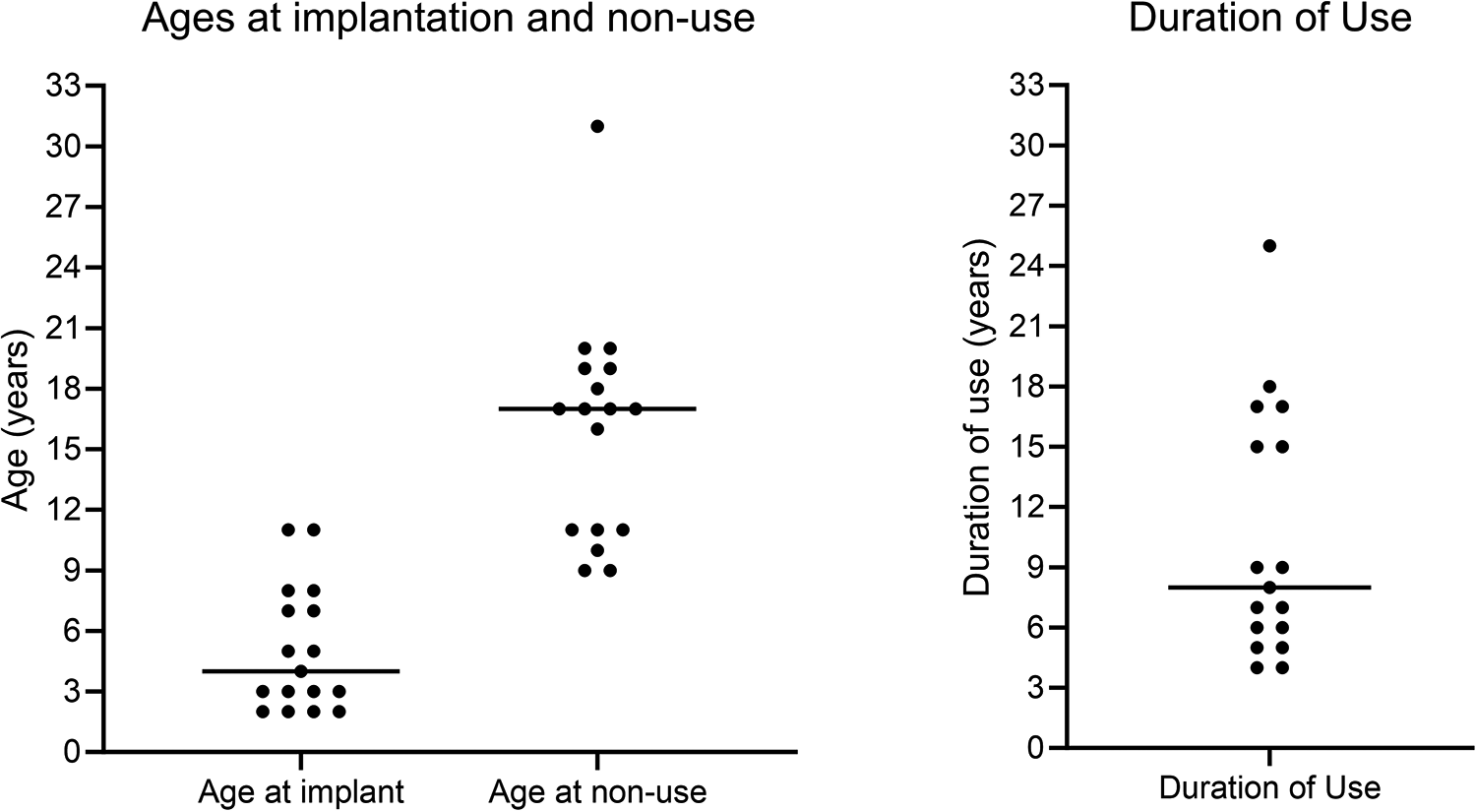
Individuals' age at first implantation, age at non-use, and duration of use.

Figure 2 shows individuals’ ages at first implantation, ages at confirmation of CI non-use, and the duration of CI use prior to confirmed non-use, in completed years. Horizontal lines show the median age or duration of use. Bimodal distributions were seen for both age at non-use and duration of use.

### 3.3. Risk factors for inconsistent or non-use

Reliance on manual communication had been identified in the literature as a risk factor for CI non-use. Table 2 documents group characteristics for both receptive and expressive communication mode, for the children and their teachers. Three of the six children reported in Table 2 to use British Sign Language (BSL) together with spoken language as their expressive communication mode were noted to either have unintelligible speech, or to use single spoken words only.

**Table 2:** Communication characteristics.

Receptive communication mode	
British Sign Language only	8 (47%)
British Sign Language and spoken	7 (41%)
British Sign Language, gesture, and spoken	1 (6%)
Gesture and spoken	1 (6%)
Spoken only	0 (0%)
Expressive communication mode	
British Sign Language only	7 (41%)
British Sign Language and gesture	1 (6%)
British Sign language and spoken^a^	6 (35%)
British Sign Language, gesture, and spoken	2 (12%)
Gesture only	1 (6%)
Sign-supported English	0 (%)
Spoken only	0 (%)
Teacher expressive communication mode	
British Sign Language only	7 (41%)
Total communication/sign bilingualism/Sign-supported English	6 (35%)
Spoken language only	1 (6%)
Unknown	3 (18%)

^a^ Three of the six children reported to use BSL and spoken language as their expressive communication mode were noted to either have unintelligible speech, or to use single spoken words only.

Table 3 presents the number of children who had each of the risk factors noted within their medical records, ranked in order of frequency across the whole group.

**Table 3:** Number and proportions of children with risk factors documented overall and by age of non-use sub-group.

Risk factor	Whole group *N* = 17	Aged <12 years at non-use *N* = 6	Aged ≥12 years at non-use *N* = 11
Primary communication mode manual	17 (100%)	6 (100%)	11 (100%)
Aged ≥3 years at first implantation	13 (76%)	5 (83%)	8 (73%)
Inconsistent CI use	8 (47%)	2 (33%)	6 (54%)
Technical or medical device issues	5 (29%)	1 (16%)	4 (36%)
Additional needs	5 (29%)	2 (33%)	3 (27%)
Complex family issues	4 (24%)	2 (33%)	2 (18%)
Poor attendance at CI appointments	4 (24%)	3 (50%)	1 (9%)
User was under peer pressure not to use CI	2 (12%)	—	2 (18%)
Family perceived no benefit from CI	1 (6%)	—	1 (9%)
Financial barriers	—	—	—

CI, cochlear implant.

**Figure 3:**
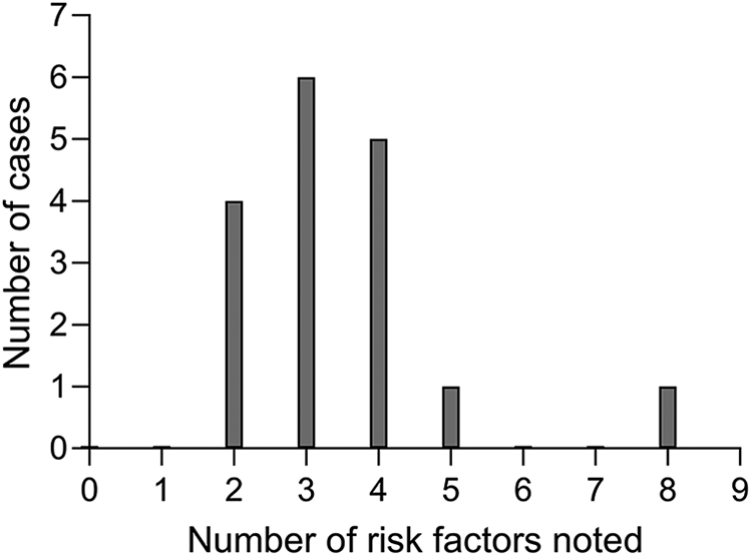
Distribution of the number of risk factors identified per child.

For each participant, the cumulative incidence of risk factors noted in their records was calculated, and the distribution of these is shown in Figure 3. Each non-user had at least two risk factors documented in their notes and most had three or four. Five of the seventeen children had additional needs. Of these, one had been implanted earlier than 2000 and four later. Children with additional needs therefore made up a larger proportion of non-users first implanted during 2000 or later (four out of seven) compared to those first implanted before 2000 (one out of ten). Of the eight non-users who were implanted under 4 years of age, half had additional needs.

### 3.4. Meaningful auditory integration scale and meaningful use of speech scale

MAIS or MUSS scores were not available for all children, nor were they recorded at the same time points across the group. Table 4 documents individuals' percentage scores on both outcomes pre-CI, and at 1 and 3 years post-CI, alongside the number of risk factors known to be present, chronological age at first CI (either < or ≥3 years of age) and age at confirmed non-use (either < or ≥12 years of age).

**Table 4:** Individuals’ cumulative number of risk factors, age category at first implantation and non-use, and parent-rated MAIS and MUSS scores.

Study Code	N Risk factors	Age at CI (years)	Age at non-use (years)	Parent-rated MAIS (Percentage scores)			Parent-rated MUSS (Percentage scores)		
				Pre-CI	1 year	3 years	Pre-CI	1 year	3 years
LT01	4	≥3	<12	—	—	—	—	—	—
LT03	4	≥3	≥12	—	92.5	97.5	—	—	45.0
LT04	3	≥3	≥12	—	—	90.0	—	—	—
LT05	3	≥3	≥12	10.0	92.5	77.5	7.5	12.5	32.5
LT06	3	≥3	<12	12.5	25.0	80.0	—	17.5	22.5
LT07	3	≥3	<12	35.0	32.5	100.0	—	10.0	—
LT10	2	≥3	≥12	20.0	17.5	80.0	10.0	12.5	40.0
LT13	8	≥3	≥12	—	—	33.0	—	—	—
LT15	2	≥3	≥12	—	—	—	—	—	—
LT16	4	<3	<12	0	12.5	17.5	—	20.0	5.0
LT17	2	≥3	<12	32.5	72.5	70.0	12.5	50.0	52.5
LT28	3	≥3	≥12	—	—	57.5	—	—	—
LT30	5	≥3	≥12	—	—	100.0	—	—	—
LT32	3	≥3	≥12	—	95.0	82.5	—	57.5	95.0
LT43	2	<3	≥12	17.5	35.0	93.0	12.5	10.0	37.5
LT44	4	<3	≥12	5.0	30.0	—	—	17.5	—
LT45	4	<3	≥12	7.5	67.5	92.5	10.0	30.0	57.5

CI, cochlear implant; MAIS, Meaningful Auditory Integration Scale; MUSS, Meaningful Use of Speech Sounds.

For the eight out of 17 patients with Parent MAIS scores available both pre-CI and at 3 years post-CI, a dependent *t*-test revealed a significant improvement over time, with mean percentage scores increasing from 16.9% to 76.3% (*p *= <.001). Similarly, for the 5 out of 17 patients with Parent MUSS scores available both pre-CI and 3 years post-CI, the mean percentage score significantly increased from 10.5% to 44.0% (*p* < .01). However, it cannot be assumed that these changes were representative of the children with missing data who could not be included in these analyses.

## 4. Discussion

This retrospective service evaluation documented the time-course from first implantation to non-use of all congenitally deaf patients implanted under the age of 18 years at a regional CI service for between 1989 and 2013. Seventeen eligible patients were identified, creating the largest group of non-users implanted as children to be described in the literature to date. Several other centers have described non-use in a minority of children. Examples include five child non-users reported by Ray et al. in 2006, representing 2.9% of their caseload (10) and four reported by Archbold et al. in 2009, representing 3% of their caseload (3). However one recent study reviewed 100 paediatric CI recipients implanted between 1992 and 2015, with 7–30 years' post-CI experience, reporting that none had become non-users (11). This apparent difference in the incidence of non-users might be due to the inclusion of all known cases in the present study, including patients who received their first CI at other hospitals and were later transferred to the service, whereas Calvino et al. (2023) included cases implanted at their center only and excluded both those who had become non-users when refusing re-implantation following explantation and those lost to follow-up. CI non-users may be inconsistent users and attenders prior to non-use (5) and may be more likely to be lost to follow-up than consistent device users. It was not possible to calculate the proportion of non-users at the service over time for comparison to other studies, due to a lack of clarity on the total number of paediatric surgeries performed, and cases transferred in or out of the service, each year during the time period involved.

In the present study, the distribution of ages at non-use was bimodal with the first cluster around 9–11 years. A group who rejected their implants around this age was also noted by Archbold et al. (2009), who identified this as being the age of entry to high school in the UK education system. The present results reinforce their argument that children may need particular support regarding CI use at that time. The follow-up period of the present study was longer than for Archbold et al. (2009), allowing the identification of a second cluster who became non-users in early adulthood, around the ages when young people in the UK leave high school and transition out of full time education. The information in hospital notes for the present study did not include age at leaving education to link to age at non-use, and no causal relationship can be assumed. However, it is important for CI providers to be aware that more intensive support might be needed for young people around such life transitions. This study identified cases where patients stopped using their CI as long as 17–25 years following first implantation, the latest documented in the literature, reinforcing the need for long-term access to rehabilitation support into adulthood.

Experience gained over the years since this cohort was implanted has led to a greater understanding of how such support can be implemented. It is the opinion of this service that parents, children, and adult CI users need ready access to rehabilitation support. A multi-disciplinary approach is favoured, including active communication between specialist staff based centrally at the CI service and local specialist professionals including teachers of the deaf and speech and language therapists. This approach promotes consistent advice-giving. As a child, the recipient needs to be placed in an education setting with staff who understand the importance of consistent speech processor use and active listening (16). Older children and young adult CI users can benefit from peer support (17). It can also be beneficial for carers to receive peer-support from families of a similar background whose children are implanted. Non-specialist local professionals such as teachers, social workers, and health visitors, can be educated by specialists to understand the long term consequences of non-attendance at appointments and inconsistent speech processor use, motivating them to intervene if necessary. Therapy tools such as Auditory Verbal Therapy (18) and the application of coaching techniques could be useful to empower and motivate adult CI users and parents of children with CIs, to optimize use and listening skills. At each stage, rehabilitation must be provided with the assistance of spoken and/or signed language interpreting support as needed. The findings of the present study highlight the importance of future investigation into the efficacy, and optimal delivery, of such services.

All non-users had at least two of the risk factors previously identified in the literature as being associated with either inconsistent or non-use. The whole group were reliant on sign language or gesture as their primary communication mode, a risk factor previously described by Archbold et al. (3). Since the first CIs were provided to congenitally deaf children, it has become apparent that there is a critical period to successfully provide auditory stimulation (19) and recent evidence shows that implantation before a child's first birthday is associated with a better chance of gaining age-appropriate spoken language (20). This was not as well understood when CIs were offered to the earliest children in this cohort. Studies such as these help clinicians provide more realistic expectation setting to children and their families during the eligibility assessment process. Each case is now evaluated by specialists who can emphasise any limitations a child is likely to experience regarding language development, speech production, and learning, so that goals are realistic. These discussions are facilitated by specialist spoken or signed language interpreters who have expertise in hearing loss where available, and language is appropriately simplified for the child who is included in discussions when possible. Frameworks similar to that proposed by Helman et al. (21), are used to aid candidate selection.

Regarding communication mode, local services providing aural/oral education and language rehabilitation were not available in every geographic area served by this service in the past. It was not possible to test for a causal relationship between communication mode pre- or post-CI and non-use in the present study. However, in line with the literature (22), earlier implantation and improved access to aural rehabilitation have developed alongside improved outcomes for this CI service over time. It is encouraging that no congenitally deaf children implanted within the later time-window included in this study, from 2007 to 2013, have yet become non-users. Further research is needed to explore the likely complex relationships between onset and degree of hearing loss, age at implantation, and communication mode, on CI non-use.

In addition to reliance on manual communication, each child also had either additional needs, chronological age of 3 years or more at first implantation, or at least one other risk factor for inconsistent CI use. However it is important to state that no causal relationships could be established and there is robust evidence to support the provision of CIs to children with additional needs (23). Only one child had been bilaterally implanted. Bilateral CIs were not funded for children in the UK until 2009, except where there was a risk of ossification following meningitis or for children with visual impairment (14). Analyses of populations where bilateral CIs had been more widely available, and the offer of bilateral CIs was not influenced by other risk factors such as communication mode, additional needs, or inconsistent appointment attendance, would be needed to establish if receiving only one CI is an independent risk factor for inconsistent or non-use.

The only category of risk factor not found for any participant in the present study was financial, as documented by other researchers regarding medical insurance (8) or the cost of replacing speech processor parts (11). This was likely due to the provision of CI surgeries, hardware, and appointments free at the point of delivery over the duration of the study via the UK's National Health Service. Future collaborative research between multiple CI services could help to clarify whether there is a relationship between the presence of particular risk factors and age at non-use.

MAIS and MUSS scores were available for an increasing proportion of the group over time. In agreement with the findings of Ozdemir et al. (2), significant improvements were seen post-CI for children with data available. Hence even children who make progress on auditory skills post-CI can later become device non-users. The MAIS scores of the four non-users identified by Ozdemir et al. (2013) fell within the wide range of scores of the present cohort. The MUSS was not reported in other studies of non-users, meaning no direct comparison could be made. However, Archbold et al. (3) found no significant association between Categories of Auditory Performance (24) scores (receptive skills) and non-use, but did find an association between non-use and expressive spoken communication assessed via the Speech Intelligibility Rating scale (25, 26). Similarly, as children, the present study group demonstrated slower progress on the MUSS than the MAIS and several were noted in their files to have unintelligible spoken communication following implantation. It is plausible that gaining some understanding of other people's spoken communication but not being able to reciprocate could have been associated with their choice to reject implant use, preferring the signed communication that had in all cases been their primary mode both before and following implantation. This hypothesis could be explored in future research.

A strength of this study was the consideration of all potentially eligible cases, with no dependence on parents or patients either completing a survey or being in contact with the clinic. However, this approach also had limitations since data were collected and recorded for routine clinical care and not for answering the research objectives. Date of non-use was defined as the date at which non-use was first recorded in the medical records. If there was a delay between true non-use and this being documented, then ages at non-use and durations of use would have been over-estimated. Changes in clinical practice over time resulted in there being missing MAIS and MUSS data in several cases, and in outcomes not being available at consistent time intervals across the group. Data on the number of children who had been implanted per year at the center prior to 2013 could not be located, preventing us from calculating the proportion of children implanted over time who subsequently became non-users, creating survival plots, or calculating the odds of a congenitally-deaf child implanted over a decade ago becoming a non-user based on different characteristics. Last, the method used in the present study does not reveal the effect that implantation had on these people and their families. Important lessons could be learned by qualitative research such as that by Salehomoum (27) into the lived experiences and opinions of CI recipients who chose not to continue CI use. This insight could help CI providers better understand, and work more effectively, with patients who present in the future with risk factors for non-use, to set expectations, plan support, and inform candidacy decisions.

## 5. Conclusion

This retrospective service evaluation described 17 congenitally deaf CI recipients implanted as children who later rejected CI use. Likely influenced by the method used to identify cases and the duration of time since first implantation, a larger group than previously reported was identified. Ages at non-use were clustered around 9–11 years or 16–20 years apart from one outlier who was older. The most commonly identified risk factors for inconsistent or non-use were reliance on manual receptive and expressive communication and chronological age of at least 3 years at first implantation. The findings support the provision of, and further research into, long-term rehabilitation support strategies, realistic expectation setting, and evidence-based CI candidacy selection for congenitally deaf children.

## Data Availability

The data analyzed in this study is subject to the following licenses/restrictions: Individuals’ characteristics data are not available to prevent any patient from being identifiable. All group data are presented within the main body of the article. Requests to access these datasets should be directed to catherine.killan1@nottingham.ac.uk.

## References

[1] Bond M Mealing S Anderson R Elston J Weiner G Taylor RS The effectiveness and cost-effectiveness of cochlear implants for severe to profound deafness in children and adults: a systematic review and economic model. Health Technol Assess (Rockv). (2009) 13(44):1–330. 10.3310/hta13440 19799825

[2] Özdemir S Tuncer Ü Tarkan Ö Kıroğlu M Çetik F Akar F. Factors contributing to limited or non-use in the cochlear implant systems in children: 11 years experience. Int J Pediatr Otorhinolaryngol. (2013) 77(3):407–9. 10.1016/j.ijporl.2012.11.041 23280278

[3] Archbold SM Nikolopoulos TP Lloyd-Richmond H. Long-term use of cochlear implant systems in paediatric recipients and factors contributing to non-use. Cochlear Implants Int. (2009) 10(1):25–40. 10.1179/cim.2009.10.1.25 18979457

[4] Raine CH Summerfield Q Strachan DR Martin JM Totten C. The cost and analysis of nonuse of cochlear implants. Otol Neurotol. (2008) 29(2):221–4. 10.1097/mao.0b013e31815c25a1 18046260

[5] Markey AL Nichani J Lockley M Melling C Ramsden RT Green KM Cochlear implantation in adolescents: factors influencing compliance. Cochlear Implants Int. (2015) 16(4):186–94. 10.1179/1754762813Y.0000000033 24624996

[6] Anmyr L Larsson K Olsson M. Parents’ stress and coping related to children’s use of a cochlear implant: a qualitative study. J Soc Work Disabil Rehabil. (2016) 15(2):150–67. 10.1080/1536710X.2016.1162123 26958933

[7] Tordrup D Smith R Kamenov K Bertram MY Green N Chadha S. Global return on investment and cost-effectiveness of WHO’s HEAR interventions for hearing loss: a modelling study. Lancet Glob Health. (2022) 10(1):e52–62. 10.1016/S2214-109X(21)00447-2 34919856 PMC8692586

[8] Wiseman KB Warner-Czyz AD. Inconsistent device use in pediatric cochlear implant users: prevalence and risk factors. Cochlear Implants Int. (2018) 19(3):131–41. 10.1080/14670100.2017.1418161 29299970

[9] Contrera KJ Choi JS Blake CR Betz JF Niparko JK Lin FR. Rates of long-term cochlear implant use in children. Otol Neurotol. (2014) 35(3):426–30. 10.1097/MAO.0000000000000243 24518403 PMC3927162

[10] Ray J Wright T Fielden C Cooper H Donaldson I Proops D. Non-users and limited users of cochlear implants. Cochlear Implants Int. (2006) 7(1):49–58. 10.1179/cim.2006.7.1.49 18792374

[11] Calvino M Sánchez-Cuadrado I Gavilán J Lassaletta L. Long-term non-users of transcutaneous auditory implants: thirty years of experience at a single institution. Int J Environ Res Public Health. (2023) 20(13):6201. 10.3390/ijerph20136201 37444049 PMC10341118

[12] Neumann K Chadha S Tavartkiladze G Bu X White K. Newborn and infant hearing screening facing globally growing numbers of people suffering from disabling hearing loss. Int J Neonatal Screen. (2019) 5(1):7. 10.3390/ijns5010007 33072967 PMC7510251

[13] Archbold S O'Donoghue G Nikolopoulos T. Cochlear implants in children: an analysis of use over a three-year period. Am J Otol. (1998) 19:328–31. 9596183

[14] National Institute for Clinical Excellence (NICE). Cochlear implants for children and adults with severe to profound deafness. NICE Technol Apprais Guid 166. (August 2008) (2009).

[15] Robbins AM Renshaw JJ Berry SW. Evaluating meaningful auditory integration in profoundly hearing-impaired children. Am J Otol. (1991) 12(Suppl.):144–50. 2069175

[16] Sanchez C Coto J Berrios D Cejas I. Impact of auditory–oral education on device use in children with hearing loss. Lang Speech Hear Serv Sch. (2022) 53(1):222–30. 10.1044/2021_LSHSS-21-00063 34958624

[17] Lasanen M Määttä K Uusiautti S. ‘I am not alone’—an ethnographic research on the peer support among northern-finnish children with hearing loss. Early Child Dev Care. (2019) 189(7):1203–18. 10.1080/03004430.2017.1371704

[18] Estabrooks W McCaffrey Morrison H MacIver-Luz K. Auditory-verbal therapy: Science, research, and practice. San Diego: Plural Publishing (2020).

[19] Harrison RV Gordon KA Mount RJ. Is there a critical period for cochlear implantation in congenitally deaf children? Analyses of hearing and speech perception performance after implantation. Dev Psychobiol. (2005) 46(3):252–61. 10.1002/dev.20052 15772969

[20] Karltorp E Eklöf M Östlund E Asp F Tideholm B Löfkvist U. Cochlear implants before 9 months of age led to more natural spoken language development without increased surgical risks. Acta Paediatr. (2020) 109(2):332–41. 10.1111/apa.14954 31350923

[21] Hellman SA Chute PM Kretschmer RE Nevins ME Parisier SC Thurston LC. The development of a children’s implant profile. Am Ann Deaf. (1991) 136(2):77–81. 10.1353/aad.2012.1077 1872264

[22] Yoshinaga-Itano C Sedey AL Wiggin M Mason CA. Language outcomes improved through early hearing detection and earlier cochlear implantation. Otology and Neurotology. (2018) 39(10):1256–63. 10.1097/MAO.0000000000001976 30444842

[23] Oghalai JS Bortfeld H Feldman HM Chimalakonda N Emery C Choi JS Cochlear implants for deaf children with early developmental impairment. Pediatrics. (2022) 149(6):e2021055459. 10.1542/peds.2021-055459 35607935 PMC9648123

[24] Archbold S Lutman M Marshall D. Categories of auditory performance. Ann Otol Rhinol Laryngol. (1995) 116(Suppl.):312–4. 7668685

[25] Allen C Nikolopoulos T O’Donoghue G. Speech intelligibility in children following cochlear implantation. Am. J. Otol. (1998) 19(6):742–6. 9831147

[26] Allen C Nikolopoulos T Dyar D O’Donoghue G. The reliability of a rating scale for measuring speech intelligibility following paediatric cochlear implantation. Otol Neurotol. 2001;22(5):631–3. 10.1097/00129492-200109000-00012 11568670

[27] Salehomoum M. Cochlear implant nonuse: insight from deaf adults. J Deaf Stud Deaf Educ. (2020) 25(3):270–82. 10.1093/deafed/enaa002 32306037

